# Book Notices

**Published:** 1894-07

**Authors:** 


### Book Notices.

An American Text-Book of the Diseases of Children. By American Teachers. Edited by Louis Starr, M.D., Physician to the Childrens' Hospital and Consulting Pediatrist to the Maternity Hospital, Philadelphia; Late Clinical Professor of Diseases of Children in the Hospital of the University of Pennsylvania, etc. Assisted by Thompson S. Westcott, M.D., Attending Physician to the Dispensary for Diseases of Children, Hospital of the University of Pennsylvania; Physician to the Out-Patient Department, Episcopal Hospital; Fellow to the College of Physicians of Philadelphia. Price: Cloth, $7; Sheep, $8; Half Russia, $9. Sold only by subscription. Philadelphia: W. B. Saunders, 925 Walnut St. 1894.

This is another of W. B. Saunders' "American Text-Books," and after examing its contents carefully, we are led to believe that it will become as popular as any of this series which have preceded it. The profession is greatly indebted to Mr. Saunders for the enterprise he has shown in publishing so many books of special merit during the past few months, and we believe it is showing a just appreciation of his efforts. These books are now among the most popular of any on the market, and there is no doubt that the demand will largely increase.

This volume is an 8vo of nearly 1200 pages, printed on excellent paper, and handsomely illustrated. The sixty-three collaborators are chosen from the most important medical centers, and all of them are teachers of the highest order. The work represents the combined efforts of these competent teachers, to each of whom was allotted his own special field of work, and the date of publication was so timed that no part of the book is out of date, as is sometimes necessarily the case where one author prepares an entire large volume.

To cover the entire field of pediatrics in a single volume, it was found necessary to condense the articles as much as possible, but this has been accomplished without any important omissions.

The classifications made in this book differ in some instances from those made in previous works on the diseases of children. The old-time classification of diarrhœal diseases into enteritis, cholera infantum and entero-colitis, has been abandoned, and they are here described as intestinal indigestion, acute milk infection, and sub-acute milk infection.

There are other changes of a minor character, most of which will increase the usefulness of the book.

The work contains besides the consideration of general diseases of children, special chapters on essential surgical subjects; on diseases of the eye, ear, nose and throat, of the skin, and on the diet. A special introductory chapter on the clinical investigation of disease and the general management of children, adds much to the value of the work.

The work is clear, concise, and has the special merit of being practical. We heartily commend it to the profession, and we believe it will prove a valuable addition to the literature of pediatrics. H.

---

A TREATISE ON HEADACHE AND NEURALGIA, INCLUDING SPINAL IRRITATION AND A DISQUISITION ON NORMAL AND MORBID SLEEP. By J. Leonard Corning, M. A., M. D., Consultant in Nervous Diseases to St. Francis Hospital; Fellow of the New York Academy of Medicine; Member of the New York Neurological Society, etc. Author of "A Treatise on Hysteria and Epilepsy," "Local Anæsthesia," "Brain Rest," etc. With an Appendix: EYE STRAIN—A CAUSE OF HEADACHE, by David Webster, M. D., Professor of Ophthalmology in the New York Polyclinic; Surgeon to the Manhattan Eye and Ear Hospital, etc., etc. Third Edition, 275 pages. Illustrated. Price, cloth, $2.75. E. B. Treat, Publisher, 5 Cooper Union, New York. 1894.

This work has met with so favorable a reception at the hands of the English speaking people that a third edition has become necessary, and in this edition some important changes have been made and much valuable matter added.

The book is divided into five parts and an appendix. Part I. treats of Headache. Pains which owe their origin to Intra-Cranial Causes. Part II., Neuralgia. Pains which owe their origin to Extra-Cranial Causes. Part III., Historical. Considerations of Methods of Treatment Heretofore Proposed. Part IV., Irritative Conditions of the Spine. Part V., Normal and Morbid Sleep. Appendix, Eye Strain, a Cause of Headache.

The various forms of headaches and neuralgias, and the other morbid conditions accompanying them, are of so much practical interest to the physician, and their cure so uncertain, that a work devoted exclusively to the consideration of these affections will be found of much benefit, and will meet with a just appreciation at the hands of the entire medical profession. It is unfortunate that we do not know more of the causation of these troubles, and that our treatment is not more certain in effecting a cure, but it is some consolation to know that progress is being made in the proper direction. Dr. Corning has given us the results of years of study and investigation in this special field of labor, and his book contains not only the latest, but the most complete and reliable information on the subject under consideration. H.

---

TREATMENT OF THE DISEASES OF THE STOMACH AND INTESTINES. By A. Mathieu, Physician to the Paris Hospitals. (Medical Practitioners' Library.) 8vo., 285 pages. Parchment muslin, price, $2.50; flexible leather, gilt top, price, $3.25. William Wood & Company, publishers, 43, 45, 47 East Tenth Street, New York.

Dr. Mathieu's connection with the Paris Hospital has given him exceptional advantages for the study of the diseases of the stomach and intestines, and the book before us bears evidence that he has made good use of the opportunities offered. This painstaking investigator is well known, and as a result of his labors in this field, this work enjoys the distinction of having a wide circulation on the continent where it is considered the best, as it is the latest, treatise upon the subject of diseases of the stomach and intestines.

The treatment of this class of diseases has undergone important changes within the past few years, and this has been brought about principally by the investigations of such men as Dr. Mathieu, and especially the study of the chemistry of digestion and the demonstration of the pathogenic importance of intoxications of intestinal origin.

It is a well known fact that for many years these diseases were treated almost entirely empirically, and not until very recent years have we begun to understand the underlying causes and how to apply rational treatment. This book will render the physician valuable assistance in the management of these diseases, and it will thus afford both him and his patients much satisfaction.

The book is elegantly bound and printed on fine heavy paper. It would be an ornament to any physicians library. H.

---

A PRACTICAL TREATISE ON NERVOUS EXHASTION (NEURASTHENIA); ITS SYMPTOMS, NATURE, SEQUENCES, TREATMENT. By George M. Beard, A. M., M. D., Fellow of the New York Academy of Medicine; of the New York Academy of Sciences; Vice-President of the American Academy of Medicine, etc., etc. Edited, with notes and additions by A. D. Rockwell, A. M., M. D., Professor of Electro-Therapeutics in the New York Post-Graduate Medical School and Hospital; Fellow of the New York Academy; Member of the American

Neurological Association, etc, etc. Third edition, enlarged. Price, in cloth, $2.75. E. B. Treat, publisher, 5 Cooper Union, New York City.

The first and second editions of this work were very popular with the profession of this country. This, the third edition, while presenting but few new facts connected with the subject of Neurasthenia, especially its causation, still there are corrections of some very grave errors that appeared in the first edition. There can be no doubt that too many diseased conditions were described under the head Neurasthenia, and as the name and the so-called disease were a popular fad, these errors were allowed to go uncorrected for too long a time. In this edition the editor is especially anxious to point out to the reader the difference between Neurasthenia and Lithœmia, the two diseased conditions having been confounded heretofore. This is a very important matter, as the two require widely different management. The one requires rest while the other demands exercise.

The editor directs particular attention to the popularity, both with the physicians and his patients, of the diagnosis, Neurasthenia, and very properly warns the profession against a too frequent use of the term. The book is filled with valuable matter, and it should have a more extended sale, as it treats from a scientific standpoint a most interesting subject. H.

---

A TREATISE ON OPHTHALMOLOGY FOR THE GENERAL PRACTITIONER. Second Edition, Revised and Enlarged, with 140 Illustrations. By Adolf Alt, M. D. Price, cloth, $3.50. J. H. Chambers & Co., Publishers. 1893.

The author has succeeded in giving everything pertaining to ophthalmology in a concise manner, yet making it perfectly lucid. It cannot fail to meet with a welcome from every practitioner, who is pushed for time, and who desires to gather the latest ideas on diseases of the eye in a plain and practical form.

The writer makes frequent references to the most practical points; and has added many ideas original with himself. The work is especially rich in practical illustrations. The subconjunctival injection of bichloride solution (1-1000) is suggested as a measure of the greatest importance in many inflammatory diseases and one which has recently come into use.

The book may be regarded as a work made from the latest teachings of the most eminent authorities; and hence upon the whole may be said to be thoroughly up to date, and may be recommended to those who wish concise information upon the newest additions on ophthalmology. H. L. H.

